# The Role of Bacterial Biofilm in Antibiotic Resistance and Food Contamination

**DOI:** 10.1155/2020/1705814

**Published:** 2020-08-25

**Authors:** Gedif Meseret Abebe

**Affiliations:** Wolaita Sodo University, College of Natural and Computational Science, Department of Biology, Wolaita Sodo, Ethiopia

## Abstract

Biofilm is a microbial association or community attached to different biotic or abiotic surfaces or environments. These surface-attached microbial communities can be found in food, medical, industrial, and natural environments. Biofilm is a critical problem in the medical sector since it is formed on medical implants within human tissue and involved in a multitude of serious chronic infections. Food and food processing surface become an ideal environment for biofilm formation where there are sufficient nutrients for microbial growth and attachment. Therefore, biofilm formation on these surfaces, especially on food processing surface becomes a challenge in food safety and human health. Microorganisms within a biofilm are encased within a matrix of extracellular polymeric substances that can act as a barrier and recalcitrant for different hostile conditions such as sanitizers, antibiotics, and other hygienic conditions. Generally, they persist and exist in food processing environments where they become a source of cross-contamination and foodborne diseases. The other critical issue with biofilm formation is their antibiotic resistance which makes medication difficult, and they use different physical, physiological, and gene-related factors to develop their resistance mechanisms. In order to mitigate their production and develop controlling methods, it is better to understand growth requirements and mechanisms. Therefore, the aim of this review article is to provide an overview of the role of bacterial biofilms in antibiotic resistance and food contamination and emphasizes ways for controlling its production.

## 1. Introduction

Food contamination by foodborne pathogens is a serious public health concern that can cause foodborne diseases [1]. Foodborne diseases are continuing to be a global public health problem with an estimated 600 million people falling ill annually [2, 3]. Food contamination may occur during any step in the farm-to-fork continuum from environmental, animal, or human sources and cause foodborne disease and intoxication [4]. Biofilm formation by foodborne pathogens is an inevitable event and becomes a source of food contamination. Bacterial biofilm formation is considered to be an emergent and prevailing microbial lifestyle in natural and manmade environments and occurs on all surface types [5, 6]. Biofilm is one of the most widespread and most successful life forms on Earth [7]. In nature, microorganisms commonly exist in the shelter of highly hydrated biofilms which creates a conducive environment for cells to adhere together and onto all kinds of surfaces [8]. Because microorganisms within this community produce cement-like matrix which can act as “biological superglue” [9], to fix or trap onto different biotic or abiotic surfaces. For instance, biofilm infections on implants or indwelling devices are difficult to eradicate because of their much better protection against macrophages and antibiotics, leading to severe clinical complications often with lethal outcome. It is a critical problem in the medical sector since it is formed on medical implants, within human tissue and involved in a multitude of serious chronic infections. Generally, biofilm is a surface-attached community of microorganisms embedded and growing in a self-produced matrix of extracellular polymeric substances [10].

Food and food processing environments are the best sites for microbial attachment and biofilm formation. Pathogenic microorganisms can attach to food surfaces, grow on them, and form a biofilm that causes an increase in the food safety risk [11]. Poor sanitation of food-contact surfaces, equipment, and processing environments has been a contributing factor in foodborne disease outbreaks, especially those involving *Listeria monocytogenes* and *Salmonella* [5]. Insufficient and ineffective cleaning practices can cause food residues to remain in food processing and can facilitate bacterial attachment and biofilm formation [6]. These surfaces with adherent microbial communities are difficult to sanitize properly since cells within a biofilm are persistent or tolerant to hygienic conditions [12]. The production of biofilm and its persistence on different surfaces related to food, medical, and other sectors would be reservoirs for many pathogens that are infectious [13]. Diverse microorganisms are able to grow on food matrixes and along with food industry infrastructures, and this growth may give rise to biofilms [14, 15]. Therefore, biofilms formed on these surfaces are the main cause of contamination of the final product. Once the biofilm is formed, then it will be hard to eradicate from these surfaces. This again could be a source of disease transmission and reduce shelf life and quality of foods [16, 17]. Furthermore, biofilm mode of growth induces microbial resistance to disinfection that can lead to substantial economic and health concerns [18]. For instance, a research done on *Listeria monocytogenes* indicates that its biocide resistance and ability to cooperate with other species forming heterogeneous communities allowed this bacterium to survive and struggle within the industrial areas [19].

Contaminated foods could be a serious problem for food quality, safety, public health, and economic impact [16]. For example, adherence to pathogens on the meat surface causes contamination of the meat, which leads to product collection from the market and causes huge economic loss at the industry and country level [2, 20, 21]. Food contaminations and foodborne diseases put their pressure on developing countries, especially in infants, children, and other susceptible communities and it also has burden on local and global markets [22]. Food contamination not only leads to economic crises but also food safety which is the primary criterion in our expanding market [23]. Therefore, illness and death from diseases caused by contaminated food are a continuing threat to public health and a major impediment to socioeconomic development worldwide [24]. Generally, the food sector is a sensitive issue that can provoke panic in the food industry if the food is contaminated.

The emergence of antimicrobial resistance is a rapidly increasing challenge in public health worldwide [25]. Biofilm-forming bacteria are embedded in a matrix and acquire properties that render them highly tolerant to antibiotics, UV light, chemical biocides, host immune response, and other external stresses [26–30]. Biofilm can protect microorganisms from harsh environmental conditions such as extreme temperature and pH, high salinity and pressure, poor nutrients, antibiotics, etc., by acting as a barrier [31]. Structural barriers, along with persistent cells within biofilm, play a decisive role in antibiotic resistance [32]. As reports indicate, biofilm-related infections are difficult for medication and will not be cured easily [33]. Consequently, the prescription of antibiotics will not solve or remove biofilm-related infection due to their antibiotic tolerance and genetic mutation [34]. Biofilm is now considered to be a primary cause of chronic infection, and antibiotic-resistant bacteria are prevalent in biofilm form [35]. Currently, it is believed that over 80% of chronic infectious diseases are caused by biofilm, and it is known that conventional antibiotic medications are inadequate at eradicating these biofilm-mediated infections [30]. As Brackman and Coenye [36] reported, antimicrobial therapy often fails to eradicate biofilm from the site of infection. Generally, antibiotic resistance has emerged at an alarming rate and becomes an escalating public health problem. This problem is amplified by biofilm formation which creates additional bacterial tolerance to antimicrobial agents [35].

The spreads of biofilm-related infections are an intractable problem in modern medicine. Biofilm formation is the main virulence factor for a wide range of microorganisms that cause chronic infections [37]. Bacterial biofilm represents a major health concern due to the high demand for implantable medical devices and the rising numbers of bacterial resistance [38]. Pathogenic microorganisms can produce biofilm on implanted devices [39]. Many bloodstream infections and urinary tract infections are associated with indwelling medical devices and arise from a bacterial biofilm that consists of bacteria embedded within an extracellular polysaccharide matrix on the catheter surface [40, 41]. For example, *Staphylococcus aureus* and *Staphylococcus epidermidis* are considered two of the most important pathogens, and their biofilm frequently causes device-associated infections [42]. According to Otto, the biofilm phenotype that these bacteria adapt during device-associated infection facilitates increased resistance to antibiotics and host immune defenses [43]. Biofilm formation by microbial pathogens enables them to survive in hosts and causes chronic infections that result in persistent inflammation and tissue damage [30]. Therefore, biofilm formation on medical instruments, human tissues, and organs has an impact on human health and the economy.

## 2. Stages of Biofilm Development

**Figure 1:**
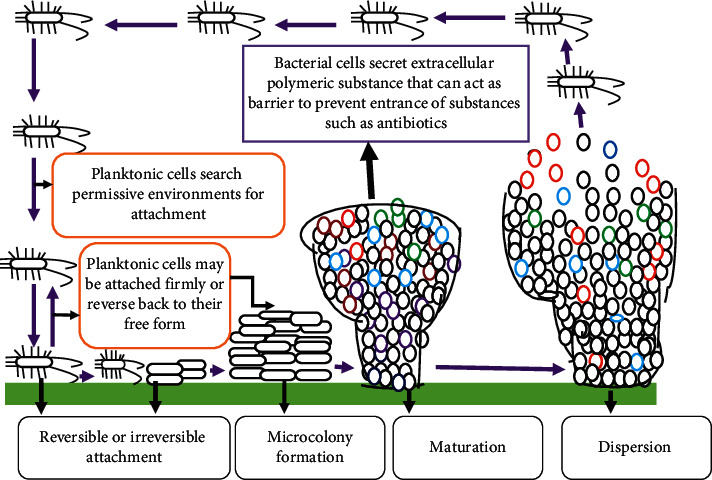
Biofilm formation and structure, adapted from [46, 47] with major modification.

**Figure 2:**
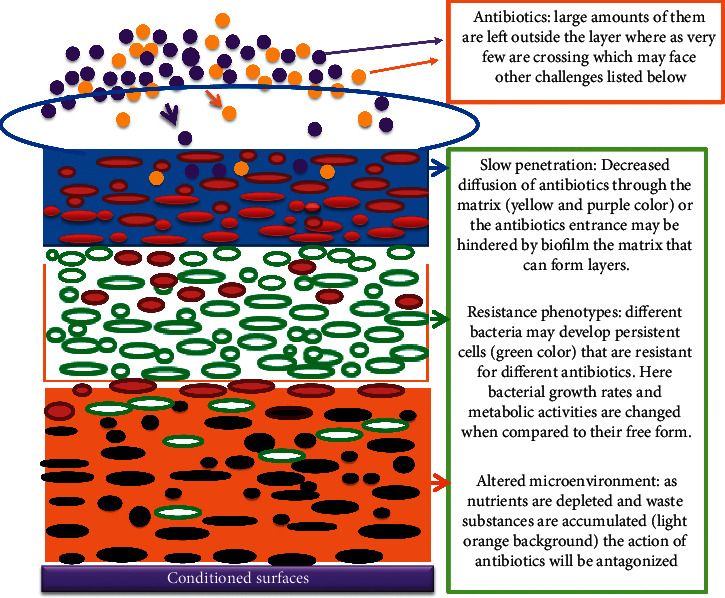
The mechanisms of antibiotic resistance in biofilms, source: [48] (with own modification).

Biofilm is an association of microorganisms that are firmly attached to the biotic or abiotic surface, encased within an extracellular polymeric substance (EPS) matrix, and that can show new character with respect to gene expression, protein synthesis, growth rate, and metabolic activities [44, 45]. Biofilm production can be influenced by a number of factors such as surface conditions, chemical and physical growth factors, cellular structures, and any other challenges. The interaction between these and other factors determines its fate [46]. As shown in Figures 1 and 2, structural and physiological change takes place after cells have been attached to conditioned surfaces. Structural polymeric substances produced are acting as a barrier [31] and prevent the entrance of antibiotics and sanitizer agents. Bacterial cell growth within biofilm is very slow and produces persistent cells that can survive hostile conditions such as exposure to antibiotics and other biocides [6, 33] (Figure 2).

Microbial cells within a biofilm are very close to each other so that they can communicate through chemicals that enable them to coordinate and respond to any ecological, environmental, and host related cues [49]. According to Oliveira et al., biofilm formation is commonly viewed as a cooperative enterprise, where strains and species work together for a common goal [50]. For this cooperative activity, there must be cell-to-cell communication. This cell-to-cell communication mechanism within the microbial community is known as quorum sensing in which microorganisms use signaling such as acyl homoserine lactone (AHL) in Gram-negative bacteria, the autoinducing peptide (AIP) in Gram-positive bacteria, and the autoinducer-2 (AI-2) in both Gram-negative and -positive bacteria for a different purpose [36, 51]. Quorum sensing (QS) system is a mechanism by which bacteria regulate the gene expression profile according to the size of the microbial population, causing the formation of different forms of biofilm [7]. As a general quorum sensing is a process by which bacteria produce and detect signal molecules and thereby coordinate their behavior in a cell-density-dependent manner [36]. In addition to communication, these close contacts microbial communities enable them to exchange genetic material, and even the frequency of gene transfer is high when compared to their free form [52]. Therefore, horizontal microbial gene transfer and biofilm formation are interrelated [53]. For biofilm formation, microorganisms should transit from their free form into a sessile form which requires stepwise physiological and structural changes [47, 54]. Thus, these stepwise and dynamical process comprises (a) initial or reversible attachment on the conditioned surface, (b) irreversible attachment (c), microcolony or early development of biofilm structure, (d) maturation of biofilm which forms mushroom or tower-like structure, and (e) dispersion or detachment in which cells slough off from the matrix and return to their original free form [47, 55] (Figure 1). Therefore, the aim of this review article is to provide an overview of the role of bacterial biofilm in antibiotic resistance and food contamination.

### 2.1. Initial or Reversible Attachment

Bacterial surface attachment represents a turning point from planktonic life to the biofilm mode [56]. Reversible attachment involves an interaction of planktonic microorganisms with a conditioned surface [57–59]. But the interaction is very weak which involves van der Waals, electrostatic forces and hydrophobic interactions. It has been reported that the attachment will be best on surfaces that are rough, hydrophobic, and coated with different organic substances [44]. Bacterial structures such as the fimbriae, pili and flagella give strength to the interaction between bacteria and the surface of attachment [60]. Generally, cell appendages involved in the reversible attachment and bacteria at this stage commit to the biofilm lifestyle or leave the surface and return to the planktonic lifestyle [56].

### 2.2. Irreversible Attachment

At this stage, loosely bound organisms consolidate the attachment process by producing extracellular polymeric substances that complex with surface materials and/or receptor-specific ligands located on pili, fimbriae, and fibrillae or both [57–59]. After microorganisms are attached on preconditioned and permissive surfaces, then the cell starts an irreversible adhesion and accumulates as multilayered cell clusters [61]. As recent studies revealed biofilm formation is commenced with a layer of polymeric substances (EPS) in which microbial cells are swarming on the surface with subsequent growth of the biofilm [62]. During this step, a number of physiological and structural changes have occurred, such as nonmotility of the attached cells [58].

### 2.3. Microcolony Formation

Microbial cells embedded within the extracellular matrix undergo coordinated community growth that leads to the formation of microcolonies. According to Dunne, microcolony formation results from simultaneous aggregation and growth of microorganisms and is accompanied by the production of EPS [57]. Microcolonies which are basic units of biofilm are compartmentalized by channels with different distinct microenvironments [29] (Figure 1). After cells are firmly attached to conductive surfaces, then numerous microorganisms will come up and secrete polymeric substances that can act as a “glue” to fix microorganisms on different surfaces. After these sequential events, microcolonies are produced.

### 2.4. Biofilm Maturation

If conditions are suitable for sufficient growth and differentiation, a biofilm may develop into spatially well-arranged, three-dimensional mature biofilm structures [61] such as mushroom or tower-like structures interspersed with fluid filled channels in which nutrients, oxygen, and essential substances can be diffused and circulate in each microenvironment [51] (Figure 1 and 2). The development of biofilm is a cooperative group behavior mediated by density-dependent chemical signals released by bacterial populations embedded in a self-produced extracellular matrix [63]. This signaling mechanism is known as quorum sensing which is used to communicate and orchestrate group behaviors, including virulence factor secretion and biofilm formation [64, 65]. Quorum sensing activates the maturation and disassembly of the biofilm in a coordinate manner [63]. Generally, cell-to-cell signaling plays a tremendous role in cell attachment and detachment from biofilm [66].

### 2.5. Biofilm Dispersal

Biofilm formation is a cyclical process in which bacterial cells are detached from the mature biofilm and enter into their previous mode of life, i.e., planktonic state. As shown in Figure 1, detached bacterial cells will seek new surfaces to attach and start up a new round of biofilm formation. In this step, microbial cells will decide based on the environmental cues whether they live together or “fall apart” [46]. From a food contamination point of view, this step is important to disseminate microorganisms into food products. Biofilm cells can be detached from actively growing cells or from the deprived environment, communication, or removal of aggregates. It has been reported that nutrient limitation forces microorganisms to seek new environments [29, 46].

## 3. Biofilm and Its Impact on Antibiotic Resistance

The emergence and spread of antimicrobial resistance among bacteria are the most important health problems worldwide [67–69]. Antibiotic resistance is one of the consequences of the bacterial biofilm communities which contribute to chronic infections [67]. Biofilm-forming *Klebsiella pneumoniae* is an important multidrug-resistant (MDR) pathogen affecting humans and a major source for hospital infections associated with high morbidity and mortality due to limited treatment options [70]. It has been reported that biofilm formation is a means for a bacterium to resist hostile environmental influences such as antibiotics and antimicrobial agents [70–73]. As Verderosa et al. reported, biofilm is recalcitrant to antibiotic therapy and a major cause of persistent and recurrent infections by clinically important pathogens worldwide [74]. This is because the formation of biofilms and subsequent encasement of bacterial cells in a complex matrix can enhance resistance to antimicrobials and sterilizing agents making these organisms difficult to eradicate and control [75–77]. The extracellular polymeric substances (EPS) matrix protects bacteria from antibiotics, avoiding drug penetration at bactericidal concentrations [38] (Figures 1 and 2). Bacteria within a biofilm are several orders of magnitude more resistant to antibiotics, compared with planktonic bacteria [78]. For instance, biofilms can tolerate antimicrobial agents at concentrations of 10–1000 times that needed to inactivate genetically equivalent planktonic bacteria [79]. As shown in Figures 1 and 2, the nature of biofilm structure and other physiological changes such as slow growth rate assists them to be resistant to antimicrobial agents [66, 80] (Figures 1 and 2). As reported microorganisms in a biofilm are resistant due to the following suggested factors: (a) polymeric matrix that can restrict diffusion of antibiotics (b) interaction of antibiotics with a polymeric matrix which lowers their activity, (c) enzyme-mediated resistance such as *β*-lactamase [73], (d) changes in metabolic activity inside the biofilm (Figure 2), (e) genetic changes on target cells or hiding the target sites, (f) extrusion of antibiotics using efflux pumps [73], and (g) the presence of outer membrane structure, such as in Gram-negative bacteria [81]. These mechanisms are critical for antibiotic resistance and survival of biofilm bacteria [73, 82]. The antibiotic resistance used by bacteria in biofilm is distinct and different from natural or innate resistance mechanisms [48] (Figure 2). As similar findings revealed bacteria within biofilm develop different molecular strategies to protect their cells from hostile conditions such as the interaction of biofilm matrix with antibiotics that can retard or lower their activities, slow growth rates in which antibiotics will not be effective, genetic related resistance, and producing persistent cells which are tolerant to different antibiotics [38] (Figure 2). In biofilm-forming bacteria, there is a high rate of mutation that enables them to develop resistant mechanisms, and this, in turn, gives an opportunity for their genes to produce enzymes that inactivate the antibiotics or expel the antibiotics using efflux pumps [34, 83]. Bacteria within biofilm produce persister cells that are metabolically inert and it is one of their mechanisms to escape from antibiotics and even they have the ability to survive in high concentration of antibiotics [84] (Figure 2). Biofilm plays a critical role in the spread of antibiotic resistance. Within the high dense bacterial population, efficient horizontal transfer of resistance and virulence genes takes place [85]. The number of microorganisms within the matrix is too dense so that there is close contact between different microorganisms which enable them to exchange resistant genes and finally, the whole community may acquire that resistant gene [68] (Figures 1 and 2). Therefore, genetic diversification of microorganisms in biofilm is largely responsible for shaping antibiotic resistance [7]. As studies have suggested that biofilm is important for the transfer of conjugative plasmids due to the high proximity of cells within this multicellular structure [86]. The resistance of biofilm to antibiotics depends on different factors such as physical, physiological, and gene-related factors [34]. Thus, this multifactorial nature of biofilm development and drug tolerance imposes great challenges for the use of conventional antimicrobials [37]. To sum up, bacterial biofilm is a key player in the development of antimicrobial resistance [38].

## 4. Biofilm and Its Impact on Food Contamination

Food contamination by pathogenic microorganisms has been a critical public health problem and a cause of huge economic losses worldwide [4]. Microbial biofilm contains both food spoiler and disease-causing bacteria and results in postprocessing contamination which lowers the quality and shelf life of products and could be a means for disease transmission [87–89]. For example, *Escherichia coli* O157 :H7 attached to beef-contact surfaces found in beef fabrication facilities may serve as a source of cross-contamination [90]. Among many pathogens, *Staphylococcu*s *aureus* and *Pseudomonas aeruginosa* are capable of constructing the biofilm on materials and equipment [91]. Friedlander et al. reported that biofilm-forming bacteria, which colonize the surfaces of equipment in the dairy industry, may adversely affect the safety and quality of the milk and its products [92]. Biofilm production by bacteria such as *Listeria monocytogenes* is supposed to be one of the ways that confer its increased resistance and persistence in the food chain [93]. The formation of biofilms on biotic and abiotic surfaces is a potential hazard, contributing to the constant circulation of pathogens in the conditions of food production and contamination of foods [94]. Pathogenic bacteria penetrate food production areas and may remain there in the form of a biofilm covering the surfaces of machines and equipment [95]. Therefore, biofilm formation by pathogenic bacteria leads to severe contamination problems in food, food processing, and other areas that directly affect human health and life [10, 96]. In a hygienic point of view, the attachment of pathogenic microorganisms to food-contact surfaces can lead to potential sanitation problems since it is persistent for long periods in hostile conditions and reservoir for contamination [16, 23, 96, 97]. In a research conducted on *Cronobacter sakazakii*, it has been reported that this bacterium is able to adhere to different surfaces such as silicon, latex, polycarbonate, stainless steel, glass, and polyvinyl chloride (PVC). Biofilm formation on stainless steel surfaces of food processing plants, leading to foodborne illness outbreaks, is enabled by the attachment and confinement of pathogens within microscale cavities of surface roughness (grooves, scratches) [98]. The attachment of microorganisms on the food preparation surface could enable microorganisms to form biofilm and become a source of contamination [87]. Generally, the growth of pathogenic bacteria such as *Escherichia coli* O157 : H7 and *Salmonella enterica* can result in cross-contamination from food processing surfaces to food products [8]. In addition to being the source of contamination, biofilms also reduce the efficiency of production and materials used in food processing [99]. Biofilms embedded in the protective extracellular polymeric substances (EPS) are difficult to remove in food production facilities [100]. Therefore, there must be appropriate methods to prevent, reduce, control, and eradicate biofilm formation on food and processing surfaces.

### 4.1. Prevention of Bacterial Biofilm Formation in Food Processing Surfaces

Biofilm has a detrimental impact on antibiotic resistance and food contamination [61]. Biofilm-forming pathogenic microorganisms are a major public health problem that is tolerant or recalcitrant to sanitizer [12, 23]. Prevention of the formation of biofilms in the industry is a crucial step in fulfilling the requirement of a safe and high-quality product. However, practically preventing or eradicating biofilm formation on food and the food processing environment once and for all is difficult [101]. For controlling the quality and safety of foods, basic governing principles must be set which aimed to follow up and check up each and every step such as Good Manufacturing Practice (GMP) and Hazard Analysis and Critical Control Point (HACCP) and Cleaning-in-Place (CIP) [29]. These principles are critical in inspecting early failures in food processing and production so that immediate action will be taken without product, economy, and time wastage. Most biofilm remediation approaches involve antibiofilm agents that target early stages of biofilm formation or biofilm dispersal agents which disrupt the biofilm cell community [74]. These agents are expected to prevent biofilm formation at the “infant” stage. For instance, the utilization of acidic electrolyzed water is aimed at disrupting microbial matrix and selected as a promising sanitizing agent in the food sector [100]. Small molecules such as antivirulence compounds, antibiofilm compounds, aryl rhodanines, chelators, N-acetylcysteine, and others can act as antibiofilm to inhibit biofilm formation [71]. Using biocontrol strategies such as bacteriocins and enzymes is considered important for the maintenance of biofilm-free systems for the quality and safety of foods [13, 102–104]. Similarly, different methods have been suggested and used to prevent and control biofilm formation such as surface modifications, cell-signal inhibition, chemical treatments, nonthermal plasma treatments, and the use of biosurfactants [13, 103]. For example, Brackman and Coenye [36] suggested quorum sensing inhibitors as promising antibiofilm agents. The other methods employed in preventing or reducing biofilm formation are disinfection. The apparatus used in the industries should be properly cleaned and disinfected, which would avoid any growth of microorganisms [23]. However, the disinfection of food-contact surfaces and environments is difficult because of sanitizer and disinfectant resistance of biofilm associated bacteria. Therefore, to overcome this problem, appropriate usage and selection of detergents and disinfectants coupled with physical methods can be suitably applied for controlling biofilm formation on food-contact surfaces [103]. There are also alternative approaches such as essential oil and bacteriophage tested as an option for the disinfection of microbial-contaminated food-contact surfaces [21, 105, 106]. A similar recommendation was also forwarded by Sadekuzzaman et al. to use novel methods and strategies which exceed conventional methods such as physical and chemical methods, sanitizers, or disinfectants, etc. [104]. As an example, antimicrobial peptides are effective and used to inhibit biofilm formation by the following mechanisms: (a) dismantling the membrane that embeds bacterial cells, (b) inhibition of their communication networks or signaling systems [107], (c) disrupting the polymeric matrix, (d) blocking the alarmone system to prevent a bacterial response, and (f) downregulating of genes critical for biofilm formation [108]. Similar findings showed that both natural compounds and synthetic analogues were used and were effective in preventing biofilm formation by quorum-quenching [76, 109].

## 5. Conclusion

Foods can be contaminated by different microorganisms and become a vehicle for foodborne pathogens and intoxication. Food contamination has been attributed to biofilms which are microbial communities living together that can be attached to biotic and abiotic surfaces. Once they attached irreversibly on these surfaces, they develop mature structures that act as a barrier against sanitizer and other agents. Consequently, they will be a source of postcontamination on later stages and resistant to harsh environmental conditions such as sanitizer. The surface in which foods can be processed must be cleaned and disinfected frequently using appropriate and effective sanitizers that can disrupt microbial cells and their attachment on food surfaces and environments. The nature of the surface in which foods can be processed is also paramount for biofilm formation. Therefore, it is better to design appropriate materials using technology which will reduce microbial attachment and conducive for cleaning. In addition to applying sanitizers and other agents, it is better also to understand their genes which are involved in encoding microbial cell surfaces that are important for attachment. The other critical issue in microbial biofilm formation is molecular cross talk or communication with their relatives by releasing signaling molecules that can alarm others for survival in hostile environments. Thus, appropriate methods should be developed to block their communication systems.

Biofilm-forming microorganisms present a serious problem in the medical sector. Biofilm-forming bacteria are encased in a matrix that enables them to exclude antibiotics and host immune response. In addition to having structural barriers, biofilm-forming bacteria can undergo physiological changes such as slow growth rate and producing persistent cells. In these occasions, antibiotics cannot inhibit, kill, or eradicate these slow-growing and persistent cells which are found inside the biofilm matrix. Therefore, chronic infections caused by biofilms are often difficult to treat effectively in part due to the recalcitrance of biofilms to antimicrobial therapy. In general, antimicrobial resistance along with biofilm formation becomes an escalating and intractable problem in the health sector and food safety.

## Data Availability

The data or information used to write this review article is available from the corresponding author upon request.

## References

[1] Akeda Y. (2015). Food safety and infectious diseases. *Journal of Nutritional Science and Vitaminology (Tokyo)*.

[2] Srey S., Jahid I. K., Ha S.-D. (2013). Biofilm formation in food industries: a food safety concern. *Food Control*.

[3] World Health Organization (WHO) (2020). *Food Safety*.

[4] Yang S.-C., Lin C.-H., Aljuffali I. A., Fang J.-Y. (2017). Current pathogenic *Escherichia coli* foodborne outbreak cases and therapy development. *Archives of Microbiology*.

[5] Lindsay D., von Holy A. (2006). Bacterial biofilms within the clinical setting: what healthcare professionals should know. *Journal of Hospital Infection*.

[6] Flemming H.-C., Wingender J., Szewzyk U., Steinberg P., Rice S. A., Kjelleberg S. (2016). Biofilms: an emergent form of bacterial life. *Nature Reviews Microbiology*.

[7] Płusa T. (2019). Znaczeniebiofilmu w kontekścienarastaniaopornościbakteriinaantybiotyki the importance of biofilm in the context of increasing bacterial resistance to antibiotics. *Pol MerkurLekarski*.

[8] Jun W., Kim M. S., Cho B.-K., Millner P. D., Chao K., Chan D. E. (2010). Microbial biofilm detection on food contact surfaces by macro-scale fluorescence imaging. *Journal of Food Engineering*.

[9] Hofkin B. V. (2011). *Living in a Microbial World, Garland Science*.

[10] Srivastava S., Bhargava A. (2016). Biofilms and human health. *Biotechnology Letters*.

[11] Hoveida L., Halaji M., Rostami S., Mobasherizadeh S. (2019). Biofilm-producing ability of Staphylococcus spp isolated from different foodstuff products. *Annali di Igiene*.

[12] Wang R. (2019). Biofilms and meat safety: a mini-review. *Journal of Food Protection*.

[13] Galié S., García-Gutiérrez C., Miguélez E. M., Villar C. J., Lombó F. (2018). Biofilms in the food industry: health aspects and control methods. *Frontiers in Microbiology*.

[14] Gutiérrez D., Rodríguez-Rubio L., Martínez B., Rodríguez A., García P. (2016). Bacteriophages as weapons against bacterial biofilms in the food industry. *Frontiers in Microbiology*.

[15] Khelissa S. O., Abdallah M., Jama C., Faille C., Chihib N.-E. (2017). Bacterial contamination and biofilm formation on abiotic surfaces and strategies to overcome their persistence. *JMES*.

[16] Shi X., Zhu X. (2009). Biofilm formation and food safety in food industries. *Trends in Food Science & Technology*.

[17] Mahdavi M., Jalali M., Kasra K. R. (2008). The assessment of biofilm formation in Iranian meat processing environments. *Research Journal of Microbiology*.

[18] Bridier A., Briandet R., Thomas V., Dubois-Brissonnet F. (2011). Resistance of bacterial biofilms to disinfectants: a review. *Biofouling*.

[19] Cabo M. L., Rodríguez-López P., Rodríguez-Herrera J. J., Vázquez-Sánchez D. (2018). Current knowledge on Listeria monocytogenes biofilms in food-related environments: incidence, resistance to biocides, ecology and biocontrol. *Foods*.

[20] Sofos J. N., Geornaras I. (2010). Overview of current meat hygiene and safety risks and summary of recent studies on biofilms, and control of Escherichia coli O157:H7 in nonintact, and Listeria monocytogenes in ready-to-eat, meat products. *Meat Science*.

[21] Giaouris E., Heir E., Hébraud M. (2014). Attachment and biofilm formation by foodborne bacteria in meat processing environments: causes, implications, role of bacterial interactions and control by alternative novel methods. *Meat Science*.

[22] Morse T., Masuku H., Rippon S., Kubwalo H. (2018). Achieving an integrated approach to food safety and hygiene-meeting the sustainable development goals in sub-saharan africa. *Sustainability*.

[23] Van Houdt R., Michielsr C. W. (2010). Biofilm formation and the food industry, a focus on the bacterial outer surface. *Journal of Applied Microbiology*.

[24] Havelaar A. H., Kirk M. D., Torgerson P. R. (2015). World health organization global estimates and regional comparisons of the burden of foodborne disease in 2010. *PLoS Med*.

[25] Nirwati H., Sinanjung K., Fahrunissa F. (2019). Biofilm formation and antibiotic resistance of Klebsiella pneumoniae isolated from clinical samples in a tertiary care hospital, Klaten, Indonesia. *BMC Proceedings*.

[26] Batoni G., Maisetta G., Esin S. (2016). Antimicrobial peptides and their interaction with biofilms of medically relevant bacteria. *Biochimica et Biophysica Acta (BBA)-Biomembranes*.

[27] Tewari A., Jain B., Dhamannapatil P. S., Saxena M. K. (2018). Biofilm resistance to antimicrobial agents and novel approaches to combat biofilm mediated resistance in bacteria, review article. *EC Microbiology*.

[28] Prakash B., Veeregowda B., Krishnappa G. (2003). Biofilms: a survival strategy of bacteria. *Current Science*.

[29] Zhao X., Zhao F., Wang J., Zhong N. (2017). Biofilm formation and control strategies of foodborne pathogens: food safety perspectives. *RSC Advances*.

[30] Li X.-H., Lee J.-H. (2017). Antibiofilm agents: a new perspective for antimicrobial strategy. *Journal of Microbiology*.

[31] Yin W., Wang Y., Liu L., He J. (2019). Biofilms: the microbial “protective clothing” in extreme environments. *International Journal of Molecular Sciences*.

[32] Estrela A. B., Abraham W.-R. (2010). Combining biofilm-controlling compounds and antibiotics as a promising new way to control biofilm infections. *Pharmaceuticals*.

[33] Hall C. W., Mah T.-F. (2017). Molecular mechanisms of biofilm-based antibiotic Resistance and tolerance in pathogenic bacteria. *FEMS Microbiology Reviews*.

[34] Ciofu O., Tolker-Nielsen T. (2019). Tolerance and resistance of Pseudomonas aeruginosa biofilms to antimicrobial agents-how P. aeruginosa can escape antibiotics. *Frontiers in Microbiology*.

[35] Bowler P. G. (2018). Antibiotic resistance and biofilm tolerance: a combined threat in the treatment of chronic infections. *Journal of Wound Care*.

[36] Brackman G., Coenye T. (2014). Quorum sensing inhibitors as anti-biofilm agents. *Current Pharmaceutical Design*.

[37] Koo H., Allan R. N., Howlin R. P., Stoodley P., Hall-Stoodley L. (2017). Targeting microbial biofilms: current and prospective therapeutic strategies. *Nature Reviews Microbiology*.

[38] Pinto R. M., Soares F. A., Reis S., Nunes C., Van Dijck P. (2020). Innovative strategies toward the disassembly of the EPS matrix in bacterial biofilms. *Frontiers in Microbiology*.

[39] Lebeaux D., Ghigo J. M., Lucet J. C. (2014). Physiopathologie et prevention des infections liées aux dispositifsmédicauximplantés implanted medical device-related infections: pathophysiology and prevention. *Review Pratice*.

[40] Donlan R. M. (2001). Biofilm formation: a clinically relevant microbiological process. *Clinical Infectious Diseases*.

[41] Aslam S. (2008). Effect of antibacterials on biofilms. *American Journal of Infection Control*.

[42] Ceresa C., Tessarolo F., Maniglio D. (2019). Medical-grade silicone coated with rhamnolipid R89 is effective against *Staphylococcus* spp. biofilms. *Molecules*.

[43] Otto M. (2018). Staphylococcal biofilms. *Microbiology spectrum*.

[44] Donlan R. M. (2002). Biofilms: microbial life on surfaces. *Emerging Infectious Diseases*.

[45] Oxaran V., Dittmann K. K., Lee S. H. I. (2018). Behavior of foodborne pathogens Listeria monocytogenes and Staphylococcus aureus in mixed-species biofilms exposed to biocides. *Applied and Environmental Microbiology*.

[46] Kostakioti M., Hadjifrangiskou M., Hultgren S. J. (2013). Bacterial biofilms: development, dispersal, and therapeutic strategies in the dawn of the postantibiotic era. *Cold Spring Harbor Perspectives in Medicine*.

[47] Welch M., Maunders E. (2017). Matrix exopolysaccharides; the sticky side of biofilm formation. *FEMS Microbiology Letters*.

[48] Stewart P. S., William Costerton J. (2001). Antibiotic resistance of bacteria in biofilms. *The Lancet*.

[49] Matz C., Flemming H. C., Wingender J., Szewzyk U. (2011). Competition, communication, cooperation: molecular crosstalk in multi-species biofilms. *Biofilm Highlights. Springer Series on Biofilms, 5*.

[50] Oliveira N. M., Martinez-Garcia E., Xavier J. (2015). biofilm formation as a response to ecological competition. *PLOS Biology*.

[51] Petrova O. E., Sauer K. (2012). Sticky situations: key components that control bacterial surface attachment. *Journal of Bacteriology*.

[52] Roberts A. P., Mullany P., Wilson M. (2004). Gene transfer in bacterial biofilms. *Methods in Enzymology*.

[53] Madsen J. S., Burmølle M., Hansen L. H., Sørensen S. J. (2012). The interconnection between biofilm formation and horizontal gene transfer. *FEMS Immunology & Medical Microbiology*.

[54] Wilkins M., Hall-Stoodley L., Allan R. N., Faust S. N. (2014). New approaches to the treatment of biofilm-related infections. *Journal of Infection*.

[55] Dufour D., Leung V., Lévesque C. M. (2012). Bacterial biofilm: structure, function, and antimicrobial resistance. *Endodontic Topics*.

[56] Toyofuku M., Inaba T., Kiyokawa T., Obana N., Yawata Y., Nomura N. (2016). Environmental factors that shape biofilm formation. *Bioscience, Biotechnology, and Biochemistry*.

[57] Dunne W. M. (2002). Bacterial adhesion: seen any Good biofilms lately?. *Clinical Microbiology Reviews*.

[58] Sauer K., Camper A. K., Ehrlich G. D., Costerton J. W., Davies D. G. (2009). *Pseudomonas aeruginosa* displays multiple phenotypes during development as a biofilm. *Journal Of Bacteriology*.

[59] Otto M. (2013). Staphylococcal infections: mechanisms of biofilm maturation and detachment as critical determinants of pathogenicity. *Annual Review of Medicine*.

[60] Jamal M., Ahmad W., Andleeb S. (2018). Bacterial biofilm and associated infections. *Journal of the Chinese Medical Association*.

[61] Roy R., Tiwari M., Donelli G., Tiwari V. (2018). Strategies for combating bacterial biofilms: a focus on anti-biofilm agents and their mechanisms of action. *Virulence*.

[62] Jang H., Rusconi R., Stocker R. (2017). Biofilm disruption by an air bubble reveals heterogeneous age-dependent detachment patterns dictated by initial extracellular matrix distribution. *Npj Biofilms and Microbiomes*.

[63] Solano C., Echeverz M., Lasa I. (2014). Biofilm dispersion and quorum sensing. *Current Opinion in Microbiology*.

[64] Kim M. K., Zhao A., Wang A. (2017). Surface-attached molecules control Staphylococcus aureus quorum sensing and biofilm development. *Nature Microbiology*.

[65] Scoffone V. C., Trespidi G., Chiarelli L. R., Barbieri G., Buroni S. (2019). Quorum sensing as antivirulence target in cystic fibrosis pathogens. *International Journal of Molecular Sciences*.

[66] Donlan R. M., Costerton J. W. (2002). Biofilms: survival mechanisms of clinically relevant microorganisms. *Clinical Microbiology Reviews*.

[67] Cepas V., López Y., Muñoz E. (2019). Relationship between biofilm formation and antimicrobial resistance in gram-negative bacteria. *Microbial Drug Resistance*.

[68] Balcázar J. L., Subirats J., Borrego C. M. (2015). The role of biofilms as environmental reservoirs of antibiotic resistance. *Frontiers in Microbiology*.

[69] Zhang J., Li W., Chen J., Qi W., Wang F., Zhou Y. (2018). Impact of biofilm formation and detachment on the transmission of bacterial antibiotic resistance in drinking water distribution systems. *Chemosphere*.

[70] Navon-Venezia S., Kondratyeva K., Carattoli A. (2017). Klebsiella pneumoniae: a major worldwide source and shuttle for antibiotic resistance. *FEMS Microbiology Reviews*.

[71] Chen M., Yu Q., Sun H. (2013). Novel strategies for the prevention and treatment of biofilm related infections. *International Journal of Molecular Sciences*.

[72] Lu L., Hu W., Tian Z. (2019). Developing natural products as potential anti-biofilm agents, Lu et al. *Chinese Medical Journal*.

[73] Høiby N., Bjarnsholt T., Givskov M., Molin S., Ciofu O. (2010). Antibiotic resistance of bacterial biofilms. *International Journal of Antimicrobial Agents*.

[74] Verderosa A. D., Totsika M., Fairfull-Smith K. E. (2019). Bacterial biofilm eradication agents: a current review. *Frontiers in Chemistry*.

[75] Khatoon Z., McTiernan C. D., Suuronen E. J., MahT -F., Alarcon E. I. (2018). Bacterial biofilm formation on implantable devices and approaches to its treatment and prevention. *Heliyon*.

[76] Satpathy S., Sen S. K., Pattanaik S., Raut S. (2016). Review on bacterial biofilm: an universal cause of contamination. *Biocatalysis and Agricultural Biotechnology*.

[77] Lajhar S. A., Brownlie J., Barlow R. (2018). Characterization of biofilm-forming capacity and resistance to sanitizers of a range of E. coli O26 pathotypes from clinical cases and cattle in Australia. *BMC Microbiology*.

[78] Rabin N., Zheng Y., Opoku-Temeng C., Du Y., Bonsu E., Sintim H. O. (2015). Biofilm formation mechanisms and targets for developing antibiofilm agents. *Future Medicinal Chemistry*.

[79] Myszka K., Czaczy K. (2011). Bacterial biofilms on food contact surfaces – a review. *Polish Journal of Food and Nutrition Sciences*.

[80] Chadha T. (2014). Bacterial biofilms: survival mechanisms and antibiotic resistance. *Journal of Bacteriology & Parasitology*.

[81] Singh S., Singh S. K., Chowdhury I., Singh R. (2017). Understanding the mechanism of bacterial biofilms resistance to antimicrobial agents. *The Open Microbiology Journal*.

[82] Ito A., Taniuchi A., May T., Kawata K., Okabe S. (2009). Increased antibiotic resistance of Escherichia coli in mature biofilms. *Applied and Environmental Microbiology*.

[83] Dzianach P. A., Dykes G. A., Strachan N. J. C., Forbes K. J., Pérez-Reche F. J. (2019). Challenges of biofilm control and utilization: lessons from mathematical modelling. *Journal of The Royal Society Interface*.

[84] Wood T. K., Knabel S. J., Kwan B. W. (2013). Bacterial persister cell formation and dormancy. *Applied and Environmental Microbiology*.

[85] Costerton J. W., Montanaro L., Arciola C. R. (2005). Biofilm in implant infections: its production and regulation. *The International Journal of Artificial Organs*.

[86] Lécuyer F., Bourassa J. S., Gélinas M., Charron-Lamoureux V., Burrus V., Beauregard P. B (2018). biofilm formation drives transfer of the conjugative element ICEBs1 in Bacillus subtilis. *mSphere*.

[87] Iversen C., Lane M., Forsythe S. J. (2004). The growth profile, thermotolerance and biofilm formation of Enterobacter sakazakii grown in infant formula milk. *Letters in Applied Microbiology*.

[88] Oh S.-W., Chen P.-C., Kang D.-H. (2007). biofilm formation by Enterobacter sakazakii grown in artificial broth and infant milk formula on plastic surface. *Journal of Rapid Methods and Automation in Microbiology*.

[89] Brooks J. D., Flint S. H. (2008). Biofilms in the food industry: problems and potential solutions. *International Journal of Food Science & Technology*.

[90] Dourou D., Beauchamp C. S., Yoon Y. (2011). Attachment and biofilm formation by *Escherichia coli* O157:H7 at different temperatures, on various food-contact surfaces encountered in beef processing. *International Journal of Food Microbiology*.

[91] Meesilp N., Mesil N. (2018). Effect of microbial sanitizers for reducing biofilm formation of *Staphylococcus aureus* and *Pseudomonas aeruginosa* on stainless steel by cultivation with UHT milk. *Food Science and Biotechnology*.

[92] Friedlander A., Nir S., Reches M., Shemesh M. (2019). Preventing biofilm formation by dairy-associated bacteria using peptide-coated surfaces. *Frontiers in Microbiology*.

[93] Lee B.-H., Cole S., Badel-Berchoux S. (2019). biofilm formation of Listeria monocytogenes strains under food processing environments and pan-genome-wide association study. *Frontiers in Microbiology*.

[94] Tutelyan A. V., Yushina Y. K., Sokolova O. V., Bataeva D. S., Fesyun A. D., Datiy A. V. (2019). Formation of Biological Films by Microororganisms in Food Productions. *VoprPitan*.

[95] Chlebicz A., Śliżewska K. (2018). Campylobacteriosis, salmonellosis, yersiniosis, and listeriosis as zoonotic foodborne diseases: a review. *International Journal of Environmental Research and Public Health*.

[96] Feng G., Cheng Y., Wang S.-Y., Borca-Tasciuc D. A., Worobo R. W., Moraru C. I. (2015). Bacterial attachment and biofilm formation on surfaces are reduced by small-diameter nanoscale pores: how small is small enough?. *Npj Biofilms and Microbiomes*.

[97] Colagiorgi A., Bruini I., Di Ciccio P. A., Zanardi E., Ghidini S., Ianieri A. (2017). Listeria monocytogenes biofilms in the wonderland of food industry. *Pathogens*.

[98] Awad T. S., Asker D., Hatton B. D. (2018). Food-safe modification of stainless steel food-processing surfaces to reduce bacterial biofilms. *ACS Applied Materials & Interfaces*.

[99] Achinas S., Charalampogiannis N., Euverink G. J. W. (2019). A brief recap of microbial adhesion and biofilms. *Applied Sciences*.

[100] Han Q., Song X., Zhang Z. (2017). Removal of foodborne pathogen biofilms by acidic electrolyzed water. *Frontiers in Microbiology*.

[101] Corcoran M., Morris D., De Lappe N. (2013). Commonly used disinfectants fail to eradicate Salmonella enterica biofilms from food contact surface materials. *Applied and Environmental Microbiology*.

[102] Kumar C. G., Anand S. K. (1998). Significance of microbial biofilms in food industry: a review. *International Journal of Food Microbiology*.

[103] Ferreira C., Pereira A. M., Melo L. F., Simoes M. (2010). Advances in industrial biofilm control with micro-nanotechnology. *Current Research, Technology and Education Topics inApplied Microbiology and Microbial Biotechnology*.

[104] Sadekuzzaman M., Yang S., Mizan M. F. R., Ha S. D. (2015). Current and recent advanced strategies for combating biofilms. *Comprehensive Reviews in Food Science and Food Safety*.

[105] Hughes G., Webber M. A. (2017). Novel approaches to the treatment of bacterial biofilm infections. *British Journal of Pharmacology*.

[106] Simo˜es M., Simo˜es L. C., Vieira M. J (2009). A review of current and emergent biofilm control strategies. *LWT-Food Science and Technology*.

[107] Yu S., Zhu X., Zhou J., Cai Z. (2018). Biofilm inhibition and pathogenicity attenuation in bacteria by Proteus mirabilis. *Royal Society Open Science*.

[108] Yasir M., Willcox M., Dutta D. (2018). Action of antimicrobial peptides against bacterial biofilms. *Materials*.

[109] Estrela A. B., Heck M. G., Abraham W. R. (2009). Novel approaches to control biofilm infections. *Current Medicinal Chemistry*.

